# Meteorological Observations Taken

**Published:** 1859-06

**Authors:** J. G. Westmoreland

**Affiliations:** The Smithsoman Institution, April, 1859, at Atlanta, Ga.


					﻿DCCQ^ooj oi^^^^a'^scccZiZiizi oc'^ !z;
t --------------------------- ---------------------------------------------------------------i
. . ... .'
cq Moqoq^z;!^:^	»2	!2i
«g» *o
(■ — - -.............— ■ ------------------------------- ----------------------------
cT i '	•	....	. .	.
MqqSs:^??;	ajocoQ ^izilziiz; oooQcnlzitzi OJ
g «_________________________ _ _	- ... __________________________
S	I '	■	.......... ...... .2
■s
« I	«w
•^1^	. oooooocoiooiooia ova looooooiooocioioooo «
A^J - §	'.................................................. t
. '^	■	g	E	®
I000c>0000icii0»0>0 0i0>0»000c>0>000000i0000	J
^g.	.	S	o
_	O	cq
____________________ ____________________ -................................... - --.^
Q -2	OS
s
qK	OOOC>C>OOIOVO>OOCVOOV3XOOOOC>>OOC>0»OOOOOC>	Q
s:/^
■C *>	r-	S
«C I '	M
&■	§§	S	12 S co O
csr^ 3	""	' i
”	xoocoeqooT(<ooooe'ic<i'*iqO'4*cqco'*coococaoe2«2:ooo^M gn
P"	Z	liatOU3'q<'q’>OCDCO:OX!l'-l'-l'^«ClO>0'^'»OOCCOt-'^'^'OcC’O.OeO'^>0
_^-CS	. eq	5;
'S ' J”___________________- _ ____ _________________________—____________________________- ®
g ®
g	sqc0»O(»oeiTj<M-xi00'f’tC0Ccqs<lO(M<MC0O'5;;J<^MONffl;*	g
O	*%*	co lO lO ‘X’	lO co	1-^ t'’ l’^ ®0 00 1'^ iO	lO iO I"- 00 I-* I**	co I?-	I'* t*	»O
ja S -^—..................  -	• ------------- - ---- -	- -	-..... -	5
S2	w a	"p
g	hj	OOOOC£)000<MCqT}<OaOOOr*OcqCOCq«OOQOOOOO^OOCC'q'022 22	&
• §	lOlOtO'rCOCO-^U3lO5Ot30OCOC£>iOxr'<ticOlOCO‘OiOO3'S<>O*O03lO'S''^
? "S '__________________________ ____________________ — - — -..............................
S^-2	•	lo-oo.oxJCcoiooc-iiooto-ooo'QOiooooocg^jiwcqoo
"Ki i	a	00 t'- 00 o » o> cq Ci	oi o o ci ci C5 o	.xi
'	.	•	^•r-'‘<-^'-icicicic>OcicJiOo’cicicic5OC:OCiciciC5CCiCiC5C0C5
p3	tp	^^ISJSl^OTwSMoqSoScocqcqc'iO'jcoeococisqcqeoeocqoqc'icqiM
I; eq ____________________________- -------------------------------------------------
ooMO<N.o>ort-oioio.o>ooioco^i«5iooq^o®o2?f7S2S
Q	W	S	li; Oi 00 00 Ci c- c >-; CM o Oi o --; -P co oo o; —_ .-< r-. c 1- Ci .-; >-• <=> co Oi	<X)
i'	•	/-<r<<-^rft^^:^LCOO:^C5OOC^CiC5C5OOOO05CSoO'CC505OO
I Q	§^^?5fJcO^S§CoScoScOCM(MOiCOCCCOCCCNCMCCCOCCOJ«^)'MC^
-g	I P5	S g i’^ 12 § S S o o o o o S S O t- 00 o o o o 1-- GO O - o co co o
Day of Mo? I
				

